# Assessment of Patient and Occupational Safety Culture in Hospitals: Development of a Questionnaire with Comparable Dimensions and Results of a Feasibility Study in a German University Hospital

**DOI:** 10.3390/ijerph15122625

**Published:** 2018-11-23

**Authors:** Anke Wagner, Martina Michaelis, Edwin Luntz, Andrea Wittich, Matthias Schrappe, Constanze Lessing, Monika A. Rieger

**Affiliations:** 1 Institute of Occupational and Social Medicine and Health Services Research, University Hospital of Tübingen, Wilhelmstraße 27, 72074 Tübingen, Germany; anke.wagner@med.uni-tuebingen.de (A.W.); Edwin.Luntz@uniklinikum-dresden.de (E.L.); 2 FFAS Research Centre for Occupational and Social Medicine (FFAS), Bertoldstraße 63, 79098 Freiburg, Germany; 3 Department of Psychosomatic Medicine and Psychotherapy, University Hospital, Hauptstraße 8, 79104 Freiburg, Germany; a_wittich@web.de; 4 Cologne Institute for Health Economics and Clinical Epidemiology, University Hospital Cologne (AöR), Gleueler Straße 176, 50935 Köln, Germany; matthias@schrappe.com; 5 Institute for Patient Safety, University Hospital of Bonn, Sigmund-Freud-Straße 25, 53127 Bonn, Germany; lessing.c@gmx.de

**Keywords:** occupational safety culture, patient safety culture, instrument development, survey, health care workers, hospital, feasibility study

## Abstract

(1) Background: Both patient and occupational safety cultures should be considered when promoting safety culture. To our knowledge, there are no studies that capture patient safety culture (PSC) and occupational safety culture (OSC) in hospitals while using a common questionnaire. The aim of this feasibility study in a German university hospital was to develop a questionnaire to assess both issues analogously. In addition to feasibility outcomes, we report results of PSC-OSC comparisons. (2) Methods: To assess PSC, we used the existing Hospital Survey on Patient Safety Culture (HSPSC) questionnaire. Developing new OSC “twin items” for certain parts of the HSPSC was supported by a previous literature review. Additionally, we developed multiple choice questions to examine knowledge and competencies regarding specific PS/OS aspects. (3) Results: Developing and implementing a combined PSC and OSC assessment instrument was feasible. The overall response rate was 33% (407 nurses, 140 physicians). In general, the statistical reliability of almost all scales was sufficient. Positive PSC perceptions (agreement rates 46–87%) were found in 16 out of 18 scales. Of the four twin scales, the PSC values were significantly better. Individual PS- and OS-related knowledge and competencies were lower than expected. (4) Conclusion: The comparative investigation of patient and occupational safety in a large hospital is a promising approach and can be recommended for further studies. We used our experiences that are presented here in an ongoing bicentric study on the associations between working conditions, occupational safety culture, patient safety culture, and patient safety outcomes (WorkSafeMed).

## 1. Introduction

In recent years, there has been increasing discussion about safety culture in the healthcare sector. A well-known definition of safety culture, which can be adapted to the healthcare sector, is provided by the Advisory Committee on the Safety of Nuclear Installations. “The safety culture of an organization is the product of individual and group values, attitudes, perceptions, competencies, and patterns of behaviour that determine the commitment to, and the style and proficiency of an organization’s health and safety management. Organizations with a positive safety culture are characterized by communications founded on mutual trust, by shared perceptions of the importance of safety and by confidence in the efficacy of preventive measures” [1]. According to this definition, safety culture can be seen as part of an organizational culture. Organizational culture therefore “represents the shared beliefs, values, attitudes, norms of behaviour of people in an organization and the established organizational routines, traditions, ceremonies and reward systems” [2].

Safety culture in publications addressing the healthcare sector often subsumes only issues of patient safety culture. Several studies in different countries have been undertaken to measure patient safety culture in hospitals. One validated and well-established instrument to measure patient safety culture is the “Hospital Survey on Patient Safety Culture” (HSPSC), as developed by Sorra & Nieva [3]. It has been used for surveys undertaken in the United States, Norway, the Netherlands, Turkey, Iran, China, Taiwan, and Japan [4,5,6,7,8,9,10]. Preliminary applications of the HSPSC have also been performed in Germany. One study consulted medical directors about the patient safety culture in their institutions [11]. In the frame of the High 5s projects [12], patient safety culture, as measured by the HSPSC, was assessed in German hospitals that implemented a surgical safety checklist [13].

In the context of a holistic safety culture, as defined above, the occupational safety of health care professionals has been little-discussed. Some approaches attempted to assess specific outcomes from insufficient occupational health in the hospital staff. All of these analyses focused one professional group only, either nurses [14,15,16,17,18] or physicians [19,20,21,22,23]. Results for both were similar, and stress, fatigue, and burnout symptoms were found to be associated with higher rates of occupational injuries and higher rates of patient-related adverse events.

Overall, there are only few studies that consider patient safety culture (PSC) and occupational safety culture (OSC) together. Initial explorations were done by the research group of Hoffman and Mark, who applied an adapted questionnaire for safety culture in industrial organisations to hospital nurses in the United States [24,25]. They found that safety culture moderated work conditions, as well as occupational injuries and patient-related adverse events. A survey of US-American nurses by Taylor et al. [26] supports these findings, showing that a poor safety culture was associated with injuries to both nurses and patients. A somewhat different focus was chosen by Halbesleben et al., who found that nurses with symptoms of burnout were more likely to have a negative perception of patient safety [27]. A recently published study in Sweden investigated, in winter 2010/2011, the relationship between patient safety climate and occupational safety climate in health care [28]. The study assessed patient safety climate and occupational safety climate with two different questionnaires and it showed a strong positive relationship between both. The authors concluded that units with a positive patient safety climate are also likely to have a positive occupational safety climate.

At a similar time (winter 2011/2012), the present feasibility study and survey were performed using a slightly different concept for the development of the questionnaire: instead of two different questionnaires, patient safety culture and occupational safety culture should be assessed through a common questionnaire as patient and occupational safety in hospitals can be seen as two sides of one coin. To our knowledge, there are no studies using this common approach. Only few studies question nurses and physicians on patient safety and occupational safety culture and compare profession-specific outcomes, although both professions work together in a team.

## 2. Materials and Methods

### 2.1. Aim of the Study

The aim of the feasibility study described here was to develop a questionnaire that captures the perceived patient safety culture and occupational safety culture of nurses and physicians and to perform a first survey in one large hospital. The data that were gathered by the survey within the feasibility study were also used to describe perceptions of both safety cultures in a large university hospital, without claiming any generalisation. The survey and results exploratively described here was part of the larger research project ABSK (“Arbeitsbedingungen und Sicherheitskultur” = Working Conditions and Safety Culture) that examined the association between front line staffs’ perceptions of working conditions, patient safety, and occupational safety culture. The main objective of the study, i.e., the feasibility of an integrated questionnaire tool, was and is the lack of studies that examine both aspects simultaneously and in both medical professional groups. The ABSK-study was conducted between 2010 and 2013.

According to Arain et al., “feasibility studies are pieces of research done before a main study” [29]. In general, the following criteria should be reported in feasibility studies according to the author:

- standard deviation of the outcome measure, which is needed in some cases to estimate sample size,
- willingness of participants to be randomised,
- willingness of clinicians to recruit participants,
- number of eligible patients,
- characteristics of the proposed outcome measure, and in some cases feasibility studies might involve designing a suitable outcome measure, and
- follow-up rates and response rates to questionnaires, adherence/compliance rates, ICCs in cluster trials, etc.

Following the description of feasibility studies according to Arain, the following topics were considered in the context of the present feasibility study on assessing patient and occupational safety culture through a common questionnaire:

- feasibility of the survey in nurses and physicians at the same time, number of eligible participants,
- response rate and respective influencing factors (“profession”, “surgical vs. non-surgical department”, and experts´ rated safety quality of the department as “low/medium/or high”),
- time needed to collect and analyse the data,
- internal scale consistency and content validity (where applicable), and
- descriptive measures of outcomes: perceived patient safety and occupational safety culture, perceived individual occupational risk and prevention, knowledge and competencies in patient safety (exemplified by the prevention of nosocomial infections in patients) and in occupational safety (exemplified by the prevention of viral infections of employees).

Further, we were interested in statistical analysis between those scales and items, which were designed to compare patient safety and occupational issues simultaneously.

### 2.2. Design, Setting and Participants

We conducted the feasibility study with a cross sectional design at a German university hospital in southern Germany, and it involved nurses and physicians who worked in inpatient care units. Operation theatres, functional units, such as ultrasound, endoscopy, cardiac catheter, and ambulance units were excluded. The medical director of the Institute of Occupational and Social Medicine and Health Services Research (MAR) recruited the department by phone contact with the respective medical director.

### 2.3. Questionnaire

The questionnaire with in total 203 items on 25 pages was designed using existing instruments where available. Generally, psychosocial working conditions should be assessed with a generic questionnaire rather than with a specific emphasis on safety culture. After the pretest, the questionnaire consisted of the following five sections and it is shown in Table 1 and Table 2.

- Patient Safety Culture (PSC): Six dimensions from the German version of the Hospital Survey on Patient Safety Culture (HSPSC) [30] were used (Table 1, dimensions nos. 1–6, slightly modified according to the German version of the questionnaire in the High 5s project [13]). Hereby HSPSC scales covering aspects that belong to the dimensions of psychosocial working conditions (e.g., scale “staffing”) were omitted in favour of generic scales derived from the questionnaire COPSOQ (e.g., scale “quantitative demands”; see below). The scale “Frequencies of events reported” (Table 1, dimension no. 7) was modified and reconstructed using own items. Two global items derived from the German Questionnaire Working Conditions in Hospitals (ArbiK) covered the general assessment of patient safety culture at the workplace and satisfaction with work processes (Table 1, dimensions nos. 8–9) [31]. All scales were operationalized following the HSPSC scheme used in the High 5s study [12,13]: Items were defined based on the five-point Likert response scale of agreement (from “strongly agree” to “strongly disagree”), or of frequency (from “always “ to “never “). For analysis, in the case of positive worded scale dimensions, we reversely coded negative worded items. Then, all answers on the five-point Likert scale were dichotomised into 0 and 1 (e.g., strongly/partly disagree, undecided vs. partly/strongly agree). In a third step, we constructed a sum score divided by the number of items. Results multiplied by 100 lead to the percentage of positive response to the dimension.
- “Twins”—Patient safety culture (PSC)/Occupational safety culture (OSC): Based on the German version of the HSPSC and the German version of the questionnaire Safety Attitudes Questionnaire (FTPS) [32], we used nine “twin”-items to analogously assess the role of hospital management and of the direct supervisor as well as the aspect of organisational learning for patient safety culture and occupational safety culture. By doing so, we acknowledged the specific importance of leadership and organisational aspects for both patient safety and occupational safety, as described in literature (e.g., refs. [33,34] for occupational safety, [35,36,37] for different aspects of both, patient and occupational safety). Items from four HSPSC scales address the direct supervisor, organisational learning, and hospital management (Table 1, dimensions nos. 15–18). Three self-constructed single items covered the perceived attitude of the direct supervisor towards PSC or OSC (Table 1, dimension no. 19, and dimensions nos. 20–21 adopted from the German version of the questionnaire Safety Attitudes Questionnaire (FTPS) [32]). One item addressed the individual’s influence on PSC and OSC at the workplace (Table 1, dimension no. 22) and another item covered a general assessment of PSC and OSC at the workplace (Table 1, no. 23). Here, items were also defined based on the 5-point Likert response scale of agreement (from “strongly agree” to “strongly disagree”, “very vast extent” to “very small extent”, and “excellent” to “insufficient, respectively). For the statistical analysis of PSC, OSC and “twins”, negatively worded items were reversed prior to calculating the scale. Agreement was determined within the relevant dimensions and was transferred to a standardised sum score, interpreted as “mean percent of agreement”.
- Occupational safety perceptions (individual occupational risk and prevention): Five self-constructed scales covered the employees´ personal perceptions of occupational risks and their responses to hazardous work situations, their attitudes towards occupational safety rules, as well as safety measures on the individual and organizational levels (Table 1, dimensions nos. 10–14). The items were developed based on a previously conducted literature review. Items were also defined based on the five-point Likert response scale of frequency (from “always” to “never”) or of agreement (from “strongly agree” to “strongly disagree”, “very important” to “very unimportant”, and “very vast extent” to “very small extent”, respectively).
- Knowledge/competencies regarding patient safety and occupational safety
  ο Patient safety (PS): Regarding patient safety, we assessed the knowledge and handling of bladder catheters following the in-house guideline of the hospital, which was updated and communicated before the survey was executed in 2012. Multiple choice questions were developed based on the content of the hospital´s guideline, depicting the two dimensions knowledge and competency: Knowledge about appropriate measures to avoid bladder catheter-associated infections (Table 2, dimension PS-know), and competency to detect and treat infections of patients with indwelling bladder catheters (Table 2, dimension PS-comp). The results of the multiple choice questions were summarised as “number of appropriate answers” score.
  ο Occupational safety (OS): Regarding occupational safety, all questions concerning the following three dimensions were derived from the German project STOP Needlestick [37]:
    ▪ knowledge about post-exposure prophylaxis in the case of exposure to Hepatitis B or C virus or HIV (Table 2, dimension OS-know),
    ▪ competency for appropriate behaviour in the case of a needlestick injury (Table 2, dimension OS-comp), and
    ▪ subjective information status to handle a needlestick injury appropriately (Table 2, dimension OS-info),

All associated items were also operationalized as sum scores to assess the number of appropriate answers.

- Structural data and psychosocial working conditions: Individual and occupational aspects included sex, age, profession, and hierarchical position in the hospital, work contract and duration of occupation details. The type of department was documented as a structure variable of the hospital. In addition, the questionnaire included nine out of 25 scales of the German version of the Copenhagen Psychosocial Questionnaire (COPSOQ) to investigate psychosocial working conditions [38]. A correlation between perceived PSC and OSC in regard to psychological stress and strain at work will be the focus of a subsequent publication. Therefore, further COPSOQ dimensions were not included in the analysis that is presented here. In addition, psychosocial working conditions were assessed with nine scales from the generic questionnaire COPSOQ (scales not shown).

The questionnaire underwent a pre-test with five physicians and 10 nurses in two hospitals of maximum care.

### 2.4. Data Collection

To assess a broad range of PSC and OSC manifestations within the feasibility study, hospital departments with different quality levels related to PS and OS management should be surveyed. Therefore, prior to the sample selection, hospital departments were subjectively rated by experts (representatives of quality management, occupational health, occupational safety department, hygiene department, and director of nursing) in a workshop. The experts were asked to assess the general quality of PS and OS management within the department. The rating was a priori predefined in three categories, named as “low/medium/or high quality” (values 1–3). In the case of differences among the experts´ assessments, only the rating of the quality management expert was considered. The recruitment was performed randomly within each of the three quality levels. At least two departments in each quality category were targeted. The rating of the eight hospital departments resulted in the following PS and OS management quality categories: One department with low quality, four departments with medium quality, and three departments with high quality.

The paper-pencil survey was conducted between December 2011 and April 2012, involving all healthcare workers in eight of 17 departments at a university hospital in southern Germany (anaesthesiology/intensive-care, paediatrics/youth medicine, neurosurgery, neurology, psychiatry/psychotherapy, radio oncology, urology, and dermatology). A member of our team (EL) visited each of the physicians’ departmental morning meetings and the handover meetings of nurses on all 65 wards. On each occasion, short oral information on the study was given and the questionnaires were distributed together with written information on the study and return envelops. Prior to the visits, we gained information about the detailed number of the staff working in the departments (physicians) and on single wards (nurses), respectively. Physicians who were absent during the specific morning meeting were addressed with a questionnaire in the personal post office box. For absent nurses, the questionnaire was stored on the ward together with written information and the return envelope. The same procedure was applied for the recall about four weeks after the first contact with nurses and physicians. Participation was voluntary, pseudonymised with anonymization by the research team immediately after receipt of the questionnaire and it included no incentives. For questionnaire return, all participants were asked to use the self-adhesive sealable return envelope. A post box was installed on the wards (for nurses) or in the departments´ secretaries (for physicians), respectively. Additionally, employees could send the questionnaire by house post or ordinary mail free of charge as postage was paid by the research team.

### 2.5. Treatment of Missing Data and Data Analysis

3–10% of the data was missing for most of the scores and single items. Four scales showed higher proportions of missing values but no relevant differences with regard the analogously verbalized “twin” scales (Table 1, no. 17: 15 (PS)/21% (OS), no. 20: 19 (PS)/18% (OS), no. 11: 22%, and no. 14: 38%). Regarding knowledge and competencies related to patient and occupational safety aspects, four out of five outcomes showed missing value rates of 5% or less, but the three items covering “Knowledge about post-exposure prophylaxis of hepatitis B, hepatitis C, and HIV- infection” were not answered by 13.7%. Missing data from scores and analysed single items were substituted by multiple imputation in IBM SPSS version 21 (25 imputations; value ranges of the original data were defined manually).

Prior to data analysis, the internal scale consistency was calculated using Cronbach´s α in the non-imputed data set. An α of 0.7 was considered as the minimum for sufficient selectivity and internal consistency [39].

Descriptive results included relative (%) and absolute frequencies, and measures of central tendency and variation (mean, standard deviation (SD), range) in the case of metric data, respectively. Likert-scaled single items are presented dichotomously in tables.

Statistical differences between answers to PSC and OSC twin scales and Likert- scaled items were calculated using the Wilcoxon-test. The significance level was 0.05 (two-sided). Effect sizes were calculated using the formula z/root_(number of valid answers)_ analogously to Cohen’s r and were categorized as ≥0.10 = small, ≥0.30 = moderate, and ≥0.50 = large effect/difference [40].

Assessing knowledge and competencies with regard to patient safety, participants of the departments “psychiatry/psychotherapy” and “dermatology” were excluded from the analysis due to little clinical relevance of the issue “indwelling bladder catheters”. This reduced the sample by 21%. Correlations between the two patient safety/occupational safety knowledge and competency outcomes were calculated using Spearman’s rho and effect size categories of ≤0.20 small, ≤0.50 medium, and >0.50 = large effect [41]. Further, results of knowledge and competencies concerning occupational safety issues were used to assess the content validity of the OSC scores by correlation analysis. The content validity was analysed by the nonparametric Kruskal-Wallis test with effect size r.

In this feasibility part of the study, physicians and nurses are described separately. Further statistical comparisons of the results will be analysed in a further publication. Response rates were calculated using two multiple logistic regression models, stratified by physicians and nurses and adjusted for type of department (surgical vs. non-surgical). Both variables were integrated simultaneous in the model, as well as experts’ rated safety quality of the department as “low/medium/or high”. Effect size parameters were odds ratios (OR), including 95% confidence interval (CI_95%_).

## 3. Results

### 3.1. Feasibility, Number of Eligible Participants and Response Rates

Three out of 11 departments, which fulfil the inclusion criteria “inpatient care”, declined to participate. They were assessed having “low” and “medium” quality with regard to PS and OS management (PS/OS quality). Eight out of 11 requested departments that were rated with different levels of PS/OS quality (high, medium, and low) agreed to participate in the study (see section “data collection”). The departments’ willingness to participate in the study did not seem to depend on different levels of PS/OS quality.

In total, the questionnaire was distributed among 1661 employees; 581 employees responded. After excluding four respondents with largely incomplete questionnaires, 13 with missing information on profession, and 17 who did not belong to either of the two medical groups, a sample of *n* = 547 was analysed (*n* = 407 nurses, *n* = 140 physicians). 409 respondents worked in non-surgical departments/wards (75%; *n* = 327 nurses, 82 physicians) and 138 in surgical departments (*n* = 80 nurses, 58 physicians).

The response rate was 32.9% (*n* = 547 of 1661) showing significant influences of the variables “Profession” and “Type of department” (surgical vs. non-surgical) in the first logistic regression model. The response rate of physicians and nurses was 29.9% and 37.0%, respectively; OR_(physicians)_ = 0.8, CI_95%_ = 0.6–0.9, *p* = 0.022; range of response rates within participating departments differed between 13.7% and 38.7% among physicians, and between 17.9% and 63.9% among nurses.

Response rates of surgical and non-surgical departments were 25.1% and 38.9%, respectively; OR_(surgical)_ = 0.5, CI_95%_ = 0.4–0.7, *p* = 0.000).

In the second regression model, the response rate was significantly higher in departments that were rated as having “high” compared to “low” PS/OS quality (reference category; 32.6% vs. 23.5%). The distance between response rates in departments with “medium” (31.7%) and “low” quality was not statistically significant.

In summary, response rates were higher among nurses, in non-surgical departments, and in departments with high PS/OS quality ratings as compared to physicians, surgical, and departments with low PS/OS quality ratings.

Characteristics of the sample (*n* = 547), as described in Table 3, differed significantly between nurses and physicians, related to occupational structure and biography. When comparing the two professions, nurses showed a higher proportion of participants with permanent employment and with part-time employment contracts; there were fewer participants with a supervising function, but more years of experience, both in occupation in the hospital and in the current department; there were also more female nurses than female physicians.

### 3.2. Time Needed to Collect and Analyse the Data

It took five months to collect all data. The data analyses required an additional three months. Research in a hospital, especially in a university hospital, is complex for a variety of reasons. First, there are many data protection requirements that must be considered. For the planned study, we required permission from the University Hospital Executive Board of Directors. In addition, the internal Data Protection Commissioner and the Staff Council was consulted. An approval from the ethics committees of the university hospital was requested, but was confirmed as not necessary for this study. Also, the personal recruitment on the 65 wards asnd in the physicians´ meetings took a lot of time, as all recruitment dates related to one department had to be coordinated in order to make sure that physicians and nurses were addressed within the same period of time. Altogether, it took five months to implement this complex scheme for recruitment and recall in all eight departments, which was partly due to the part-time job of the team member responsible for this task.

### 3.3. Internal Scale Consistency and Content Validity of Scales and Expert Ratings

Constructing “twin” items and operationalizing competencies and knowledge to measure their correlation with patient and occupational safety aspects was successful. The statistical reliability of all HSPSC and almost all adapted HSPSC scales was sufficient (Cronbach´s α = 0.71–0.89), with the exception of the two scales “Direct supervisors’ expectations and actions promoting PS/OS” (Table 1, no. 15 with Cronbach´s α = 0.59 (PS) and α = 0.61 (OS), respectively). All other twin scales showed comparable results. Sufficient values were also found in the three remaining scores indicating personal perceptions of occupational risks and their prevention (nos. 10–12; Cronbach´s α = 0.74–0.82).

Assessing the content validity of the four OSC twin scales, slight positive correlations between them and OS knowledge and competencies (see Table 2) were found for three scales, namely:

- between OS knowledge and “Organisational learning–continuous improvement of OS” and “Hospital management’s support for OS” (nos. 17 and 18 in Table 1; both rho = 0.13, *p* = 0.003), and
- between OS competencies and “Direct supervisor’s expectations and actions promoting occupational safety” (nos. 15; rho = 0.13, *p* = 0.003).

For the fourth twin “Direct supervisors’ support for OS”, the correlation result just missed the significance threshold. No correlation could be found between competencies and knowledge and other occupational safety scores.

The content validity of the experts’ rating of the quality of PS and OS management in the participating departments, as compared to the employees’ general assessment of PS and OS at the workplace (global item) can be described as follows: In departments with “low” quality as assessed by the experts, the employees’ assessment of the patient safety culture was also slightly, but significantly worse as compared to departments with medium or high quality according to the experts´ ratings (CC = 0.17, *p* = 0.003). We found the same tendency for occupational safety, but without significance at 5% error probability level (CC = 0.13, *p* = 0.065). 10.2% of all respondents judged the general patient safety as “insufficient or inadequate”, 47.5% as “acceptable”, and 47.5% as “very good or excellent”. Occupational safety was assessed much worse (12.6, 53.4 and 34.0%; CC = 0.56, *p* = 0.000).

Looking into depth, five out of 12 PSC scales showed comparable significant effects, among them two twins: “Non-punitive response to error”, “Communication openness (organizational learning)”, “Teamwork within units”, “Direct supervisor’s expectations and actions promoting patient safety”, and “Direct supervisors’ support for patient safety” (nos. 1, 5, 6, 15, and 16 in Table 1). Effect sizes were medium or high (r = 0.44/0.30/0.30/0.62/0.39; *p* = 0.006/0.031/0.029/0.001/0.011 in the previous order).

Concerning adapted OSC scales, the same tendency was true for two out of four twins (“Direct supervisor’s expectations and actions promoting occupational safety” and “Direct supervisors’ support for occupational safety” (nos. 15 and 16 in Table 1; r = 0.39/.33; *p* = 0.011/0.022). Out of further five self- constructed OSC dimensions, the effects were found only for “Subjective assessment of specific protective measures (behaviour and regulations) related to infectious diseases” (no. 13; r = 0.39; *p* = 0.011).

### 3.4. Perceived Patient Safety Culture (PSC)

**Figure 1:**
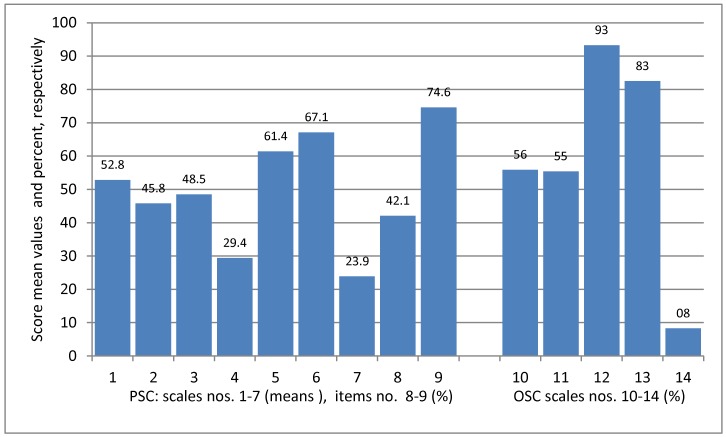
Patient safety and occupational safety culture (PSC and OSC): Results of non-”twin” scales and items (average percent of agreement to positive coded items).

For most PSC scales, between 46% and 67% of those questioned were in line with a positive patient safety culture (Figure 1, nos. 1–3, 5, 6). The scale “Feedback and communication about errors” and the item “Frequencies of events reported” were rated comparatively less positive (nos. 4 and 7). However, the variance of answers was high (see legend of Figure 1), and agreement was not statistically associated with surgical or non-surgical type of department. The global item “Trustworthiness of work unit” (no. 9) was rated positively with an agreement of 75%, while the global item “Satisfaction with work processes” (no. 8) received only 42% of agreement.

**Figure 2:**
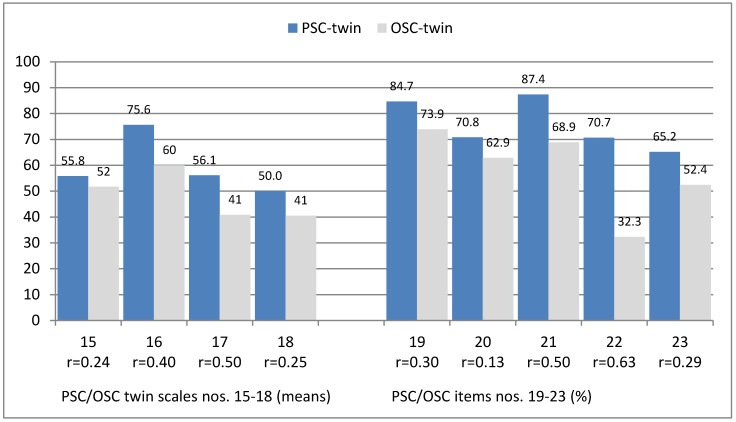
Patient and occupational safety culture (PSC and OSC): Results of “twin” scales and items (average percent of agreement to positive coded items).

Results of the remaining four PSC scales, which were designed as twin scales, can be derived from Figure 2. The highest agreement was found for the HSPSC twin scale “Direct supervisors’ support for PSC” (no. 16), followed by values between 41–60% with large variance.

Three out of six single twin-items were related to PSC attitudes and behaviour of supervisors, one to the individual influence on PSC at the workplace (nos. 19–22) and one global item addressed PSC (23). In general, all six items were rated positively—with an agreement of between 65% and 87%, particularly with “Direct supervisors’ addressing of problems” (no. 19) and “Direct supervisors’ attention to PSC” (no. 21).

Legend: Please note: PSC and OSC-scale “means” = calculated as means of percent of agreement to the single items of the scale. /Data range was always 0–100 (exception: no. 7 “Frequencies of events reported”). PSC and OSC were assessed separately with scales verbalized analogously covering either PSC or OSC.

Dimensions of patient safety culture (PSC), scales nos. 1–7 (standard deviations (SD) in brackets):

(1) Non-punitive response to error (SD 37.2)
(2) Overall perceptions of safety (SD 35.7)
(3) Hospital handoffs and transitions (SD 32.4)
(4) Feedback and communication about errors (SD 28.1)
(5) Communication openness (SD 36.3)
(6) Teamwork within units (SD 34.9)
(7) Frequencies of events reported (SD 24.6)

PSC single items nos. 8–9 (% agreement partly/fully):

(8) Satisfaction with work processes (global item)
(9) Trustworthiness of work unit (global item)

Occupational safety perceptions (perceived individual occupational risk and prevention):

(10) Personal perception of the frequency of occupational risks (SD 28.6)
(11) Attitudes towards occupational safety rules (SD 26.5)
(12) Subjective assessment of occupational safety measures initiated by the employer, related to own safety (SD 15.8)
(13) Subjective assessment of specific protective measures (behaviour and regulations) related to infectious diseases (SD 20.4)
(14) Frequency of contact to responsible specialist /official after hazardous work situations (SD 14.8)

Legend: Please note: PSC and OSC-scale “means” = calculated as means of percent of agreement to the single items of the scale. Data range was always 0–100. Effect size categories r_(Wilcoxon-test)_: ≥0.10 = small, ≥0.30 = moderate, and ≥0.50 = large effect/difference.

Dimensions of PSC and OSC twin scales nos. 15–18 (standard deviations (SD) in brackets):

(15) Direct supervisor’s expectations and actions promoting safety (28.4/30.3)
(16) Direct supervisors’ support for PSC/OSC (33.4/37.8)
(17) Organisational learning–continuous improvement of PSC/OSC (36.7/36.6)
(18) Hospital managements’ support for PSC/OSC (40.7/40.4)

PSC and OSC items nos. 19–23 (all items were verbalized analogously assessing either PSC or OSC):

(19) Direct supervisor’s addressing of problems related to PS/OS- aspects
(20) (Direct supervisor’s increased focus on PSC/OSC
(21) Direct supervisors’ attention to PSC/OSC
(22) Individual influence on PSC/OSC at the workplace
(23) General assessment of PS/OS at the workplace (global item; excellent/very good)

### 3.5. Perceived Occupational Safety Culture (OSC)

With a wide range of variation and analogous to PSC results, agreement for the twin scales nos. 15 and 16 was between 52% and 60% (“Direct supervisors’ expectations and actions promoting safety” and “Direct supervisors’ support for OSC”; see again Figure 2). Lower values than average were found for “Organisational learning—continuous improvement of OSC” and “Hospital managements’ support for OSC” (nos. 17 and 18).

Three single items that are related to the attitudes and behaviour of supervisors toward OSC—analogous to PSC results—were rated positively with an agreement of between 63% and 74% in the cases of direct supervisors’ “...addressing of problems”, “...increased focus on OSC”, and “…attention to OSC” (nos. 19–21). The items “Individual influence on...” and “General assessment of OSC at the workplace” were rated lower (nos. 22–23).

### 3.6. Occupational Safety Perceptions (Individual Occupational Risk and Prevention)

On average, over half of those surveyed perceived the frequency of occupational risks as “often/always” (i.e., exposure to infectious agents or dangerous substances, fear of skin disease, and consequences of extended work shifts, see again Figure 1 no. 10). This was similar for positive attitudes towards different occupational safety rules (no. 11).

A high agreement (>80%) was found for “Subjective assessment of occupational safety measures initiated by the employer, related to own safety” and “Subjective assessment of specific protective measures related to infectious diseases” (nos. 12 and 13). On the other hand, agreement was very low for “Frequency of contact to responsible specialists /officials after hazardous work situations” (no. 14). Standard deviations were high, with two exceptions: “Subjective assessment of occupational safety measures” (no. 12) and scale no. 14, as mentioned previously. In the case of scale no. 10 (“Perceived frequency of occupational risks”), the variance of answers was determined by the highest risk perception addressing extended work shifts, and the lowest addressing hazards of skin disease.

### 3.7. Knowledge and Competencies about Patient and Occupational Safety

The two sum scores of correctly answered items to assess individual knowledge of patient safety (“PS-know”) and competencies (“PS-comp”) were 4.1 and 2.2, respectively (SD 1.0 and 0.5, possible range 0–6 and 0–3; see Table 4). In total, 14% and 24% of the respondents answered all multiple choice items to assess patient safety knowledge and competencies correctly (see right column).

The corresponding occupational safety score values OS-know and OS-comp are also shown in Table 4. Responses regarding “knowledge about appropriate post-exposure prophylaxis of hepatitis B and C virus and HIV infection” were comparably high: 2.0 ± 0.6 (possible range 0–3). Scores measuring the “competency for appropriate behaviour in the case of a needlestick injury” were very high: 4.6 ± 0.7 (possible range 0–5). 14% (OS-know) and 67% (OS-comp) of the respondents correctly answered all of the multiple choice items. 78% of the respondents felt well-informed “to treat a needlestick injury appropriately” (“OS-info”).

### 3.8. Statistical Comparisons Between Occupational Safety and Patient Safety

Differences between occupational safety culture and patient safety “twins”: In summary, the respondents agreed significantly less to all occupational safety culture as compared to the respective patient safety culture “twin” (see again Figure 2). The effect sizes were moderate to high (exception: low effect size for no. 20).

No significant correlations were found between PS and OS knowledge and competency scores. The same was true when comparing the two PS scores with each other. A low correlation was found between occupational safety knowledge and competency scores (OS-know/OS-comp; rho = 0.13, *p* = 0.000) in the total sample.

## 4. Discussion

The study dealt with the feasibility of developing and using a questionnaire covering patient safety (PS) and occupational safety (OS) in hospitals analogously. In addition, we report the results of descriptive and inferential statistics to detect differences between two medical professional groups in a university hospital within an explorative approach. According to the design of the study, the results may not be generalised, but they will serve as a starting point for further studies. However, it seems justified to present and discuss the content-related results and to reflect on the methodological aspects of the feasibility study. In the following, the results will be discussed along with the initially formulated topics. Important issues regarding the development of the questionnaire and the feasibility of the survey, as described e.g., by Arain et al. [29], can be considered.

### 4.1. Feasibility, Number of Eligible Participants and Response Rate

The methodological approach of the study focused on assessing the perception of patient and occupational safety, as well as the respective knowledge and competencies of physicians and nurses. This approach proved to be feasible. Using published PS-specific instruments (mainly the HSPSC questionnaire) as a basis for constructing OS-specific items and scales was a good concept and produced data, which could be well analysed comparatively.

The items and scales, statistical measures (means, standard deviations) of them, as well as the differences that were described between physicians and nurses showed their general suitability and can serve as a basis for the selection of outcome variables in further (interventional) studies.

The willingness of eight out of eleven departments to participate in the study was relatively high and did not seem to be associated with their different levels of PS/OS quality, as rated in advance by experts.

Response rates were higher for nurses (41–70%) than for physicians (25–28%), but were similar to those in other studies with similar topics (41–70% for nurses and 25–28% for physicians, respectively [42,43,44]). Higher rates in non-surgical departments might have reasons in less time pressure as compared to surgical departments. Lower rates in departments with “low” PS/OS quality ratings of experts might be correlated to less consciousness of employees and therefore lower perceptions of the relevance of the topic to answer. Of course, the unequal distribution of PS/OS quality in departments and also of job-related differences in response rates reflects the biased sample. With regard to the character of the feasibility study, these are valuable findings to be borne in mind when developing the sampling strategy in future studies.

### 4.2. Internal Scale Consistency and Content Validity of Scales and Expert Ratings

The internal consistency of the HSPSC scales that were derived from the High 5s project and the related OSC-twins, as well as the adapted HSPSC scales and the related OSC-twins, was sufficient. The scales “Communication openness” and “Organisational learning—continuous improvement of PS” showed better reliability values compared to other collectives in Germany, Switzerland, or France [11,12,13,14,15,16,17,18,19,20,21,22,23,24,25,26,27,28,29,30,45], as a recent overview of HSPSC psychometric properties in European studies verified [46].

Content validity of the OSC twin scales could be confirmed by correlations with measured knowledge and in one case competency concerning occupational safety aspects. The lack of correlation of the further OSC dimensions, which we considered to be suitable for the measurement of occupational safety culture, limits the quality of the self-developed instrument. Also, these further dimensions could not confirm the content validity of the experts’ rating of OS management quality in the participating departments. Therefore, in future studies, the “non-twin” scales and items that were verbalized to capture OSC must be developed further.

### 4.3 Perceived Patient Safety Culture (PSC)

Positive PSC perceptions with consent rates averaging 46–87%, but with high standard deviations, were found in 16 out of 18 scales (non-twin scales and twin-scales). Most of the scales showed more positive results than those in studies from other northern European countries [47,48]. Overall, the results are comparable to the German High 5s study [13]

The low values found for “Feedback and communication about errors” and “Frequencies of events reported” (Figure 1, nos. 4 and 7) were not comparable to the results of the German High 5s study [13] or to the results from another comparable project [49]. Feedback and communication about errors and reporting about unexpected events are crucial elements for a patient safety culture, as they show how health professionals deal with errors.

Despite the restricted explanatory power of unanimous subjective answers and the lack of generalizable information from having just one hospital in our study, our results still imply a great need for continual improvement of patient safety issues [50]. In addition, the HSPSC proved to be a valuable instrument for the assessment of PSC.

### 4.4. Perceived Occupational Safety Culture (OSC)

Based on the four twin scales, values of occupational safety culture were rated significantly lower when compared to respective patient safety culture PSC scales. These results were not comparable to a study conducted in Sweden, which showed that patient safety climate and occupational safety climate were strongly positively related to each other [28]. The authors concluded that units with a high patient safety climate are also likely to have a high occupational safety climate. In our sample, PSC was rated higher than OSC, which might be explained by professional attitudes attaching a higher importance to the care for patients than to the employees’ own health and safety. However, this is just one assumption in the frame of a feasibility study and further studies should be undertaken to investigate patient safety culture and occupational safety culture together. In summary, the results of this study support the need for future studies on OSC in hospitals and with a multi-professional perspective.

### 4.5. Occupational Safety Perceptions (Individual Occupational Risk and Prevention)

Considering OSC aspects predominantly on the individual level, it is remarkable that, on average, only half of the investigated hospital staff perceived the frequency of occupational risks “often/al-ways”. The exposure to various physical and psychomental risks are well known [51,52,53] and they were also verified by e.g., Wicker et al. [54]. In 2008, they reported 31% out of 720 German university hospital respondents experienced a needlestick injury in the last 12 months. It might be assumed that after years of occupational risk exposure, a kind of cognitive dissonance reduction occurs [55].

On average, only half of the respondents in the current study had positive attitudes towards different occupational safety rules. These results are suggestive and they imply that hospitals should support strategies for nurses and physicians to improve and train occupational safety. A successful example of strengthening awareness of occupational risks and practical solutions was the German STOP Needlestick project [37].

### 4.6 Knowledge and Competencies about Patient Safety (PS) and Occupational Safety (OS)

The low adequate PS knowledge concerning bladder catheters was surprising, because an updated hospital guideline addressing this topic was implemented just prior to the study. While the quality management expert recommended us to use this outcome, we later found out that there was no information available on how the new guideline was actually implemented in the departments, i.e., if there were any other activities than publication and dissemination of the new guideline. The results of our survey suggest that at the time point of our study the new guideline was not yet implemented comprehensively into daily routines.

Although a majority of respondents subjectively reported a positive knowledge level for treating a needlestick injury appropriately, only a minority showed sufficient knowledge related to occupational safety about post-exposure prophylaxis of hepatitis B- and C-virus infection and HIV. Other studies in this field also revealed deficits regarding the respective aftercare [37,56,57], finding the same gap between subjective statements and tested knowledge. Interestingly, more respondents reported the sufficient competency of appropriate behaviour in the case of needlestick injuries.

We found no significant correlations between the outcomes with regard to PS (i.e., PS knowledge and PS competencies), between the outcomes describing PS and OS knowledge, or between the respective outcomes with regard to PS and OS competency. However, there was a significant, although only weak correlation between OS knowledge and OS competencies, especially in nurses. One explanation for these findings could be that the content of the updated in-house guideline for preventing nosocomial infections in patients with indwelling bladder catheters was not yet well enough disseminated at the time point of the survey, whereas the knowledge and competencies with regard to OS were better established.

Given the background that shortcomings in patient and occupational safety issues are already detectable among medical and nursing students [58,59], and to summarize our findings, it seems necessary to intensify the relevant issues from the beginning of vocational training [50,60], as well as later during continuing education and further training of professionals [60,61]. A helpful basis might be the recommendations of the German Coalition for Patient Safety, which has published the “Pathways to Patient Safety” providing knowledge and skills on patient safety for health professions [61].

### 4.7. Limitations

There are several of limitations in this study. First, the study was carried out in only one hospital, which was sufficient for a feasibility approach. Results should not be regarded as representative for hospitals on the national or international level possibly due to different frame conditions in a teaching hospital and also to the questionnaire response rate of only 33% with more nurses than physicians participating. Generally, the value of this feasibility study presented here should be that these specific observations and sources of bias should be born in mind in future studies.

Second, since no non-responder analysis was performed, we cannot fully rule out systematic differences between our study population and non-responders.

Third, our questionnaire covered only self-reports. In order to comprehensively measure knowledge and competences appropriately, other methods, such as observation, should be used. In addition, a qualitative research approach would also be conceivable to depict more detailed experience with regard to patient and occupational safety.

Fourth, the content validity might be insufficient, especially of the items to assess PS knowledge and PS competencies and of the “non-twin” OSC items. The cross-sectional design did not include randomisation and power analysis, although it did follow the phased approach to the development and evaluation of complex interventions of Campbell et al. [62] addressing the “theory” and “modelling” phases. Due to possibly different perceptions, knowledge and competencies of nurses and physicians, total results are not detailed enough and they should be presented in a further publication.

In summary, the study was carried out more than five years ago with a cross-sectional design. It cannot be ruled out that the views of physicians and nurses on patient and occupational safety have changed in the meantime. However, patient and occupational safety remain key challenges for hospitals and improvements to these areas are still required. The development and implementation of good research practices in support of these actions remains a key task for scientists in the field of occupational and public health.

Authors should discuss the results and how they can be interpreted in the perspective of previous studies and of the working hypotheses. The findings and their implications should be discussed in the broadest context possible. Future research directions may also be highlighted.

## 5. Conclusions

The results of the ABSK study presented here indicate that the comparative investigation of patient safety and occupational safety in hospitals within a multi-aspect approach addressing culture, knowledge, and competencies might be promising with regard to

- how such a study could be performed well (including sampling and recruitment strategy, sample size calculation, and non-responder analysis),
- the development and evaluation of measurement tools for the simultaneous assessment of patient safety culture and occupational safety culture, and
- the investigation of patient-related outcomes in conjunction with both patient safety and occupational safety cultures in a larger sample in order to describe relevant predictors.

Improvements related to patient and occupational safety can be derived from the results of our study. However, our study can only be considered as a starting point for further studies. There is a great need for further studies to better understand the findings that are described here and to address other phases to develop and evaluate complex interventions.

The experiences from this feasibility study were the basis for an ongoing bicentric study on associations between working conditions, occupational safety culture, patient safety culture, and patient safety outcomes (WorkSafeMed [63]), which was funded by a grant from the German Federal Ministry of Education and Research (BMBF, FKZ: 01GY1325A/B).

## Figures and Tables

**Table 1:** Patient safety culture (PSC) and occupational safety culture (OSC): Scales and items.

No.	Dimensions	Items	Response Categories	Source
		**Patient Safety Culture (PSC)**		
1	Non-punitive response to error	Please assess your unit:	Strongly/partly agree–undecided–partly/strongly disagree	HSPSC (High 5s)
		1.Staff feel like their mistakes are held against them 2. When an event is reported, it feels like the person is being written up, not the problem 3. Staff worry that mistakes they make are kept in their personnel file		
2	Overall perceptions of safety	Please assess your unit:	Strongly/partly agree–undecided–partly/strongly disagree	HSPSC (High 5s)
		1. Patient safety is never is never sacrificed to get more work done 2. It is just by chance that more serious mistakes don’t happen around here 3. We have patient safety problems in this unit 4. Our procedures and systems are good at preventing errors from happening		
3	Hospital handoffs and transitions	Please indicate to what extent you agree with the following statements about your unit:	Strongly/partly agree–undecided–partly/strongly disagree	HSPSC (High 5s)
		1. Things “fall between the cracks” when transferring patients from one unit to another 2. Important patient care information is often lost during shift changes 3. Problems often occur in the exchange of information across hospital units 4. Shift changes are problematic for patients in this hospital		
4	Feedback and communication about errors (organizational learning)	How often do you encounter the following situations in your unit?	Always—often—sometimes—seldom–never	HSPSC (High 5s)
		1. We are given feedback about changes put into place based on event reports 2. In this unit, we discuss ways to prevent errors from happening again 3. We are informed about errors that happen in this unit		
5	Communication openness (organizational learning)	How often do you encounter the following situations in your unit?	Always—often—sometimes—seldom–never	HSPSC (High 5s)
		1. Staff feel free to question the decisions or actions of those with more authority 2. Staff will freely speak up if they see something that may negatively affect patient care 3. Staff are afraid to ask questions when something does not seem right		
6	Teamwork within units	Please assess your unit:	Strongly/partly agree–undecided–partly/strongly disagree	HSPSC (High 5s)
		1. People support one another in this unit 2. When one area in this unit gets really busy, others help out 3. When a lot of work needs to be done quickly, we work together as a team to get the work done 4. In this unit, people treat each other with respect		
7	Frequencies of events reported	If a critical incident (e.g., a near-mistake) occurs in your unit—how often is it reported as critical incident...	Always—often—sometimes—seldom–never	Adapted from HSPSC (High 5s)
		1. if it (e.g., a mistake) is noticed and corrected before the patient is affected? 2. if it (e.g., a mistake) occurs which could potentially harm the patient, but does not? 3. if it (e.g., a mistake) occurs which harms the patient?		
8	Satisfaction with work processes (global item)	I am very satisfied with the way work processes are organized in our unit	Strongly/partly agree–undecided–partly/strongly disagree	ArbIK
9	Trustworthiness of work unit (global item)	Without any reservations, I can recommend our unit to potential patients	Strongly/partly agree–undecided–partly/strongly disagree	ArbIK
		**Occupational Safety Culture (OSC)**		
10	Personal perception of the frequency of occupational risks	1. How often does a situation, which is hazardous for you, occur in your hospital? 2. Do you feel exposed to hazards of infection? 3. Do you feel exposed to hazards of skin disease? 4. Do you feel exposed to serious consequences of extended work shifts? 5. Do you feel exposed to hazardous substances?	Always—often—sometimes—seldom–never	Self-constructed item(s)
11	Attitudes towards occupational safety rules	How much do you value the following measures concerning your own safety and health at work?	Strongly/partly agree–undecided–partly/strongly disagree	Self -constructed item(s)
		1. The adherence to work safety regulations should be controlled more rigidly 2. Violations of work safety regulations should lead to clear consequences 3. Work safety regulations make work in hospitals safer 4. Work safety regulations can sometimes be constricting		
12	Subjective assessment of occupational safety measures initiated by the employer, related to own safety	How much do you value the following measures concerning your own safety and health at work in the hospital:	Very important –important—partly—unimportant—very unimportant	Self -constructed item(s)
		1. regulations on how to act in the case of fire or other emergency, 2. escape and emergency exits, 3. procedures after a work accident, 4. first aid organization, 5. work time/shift regulations, 6. instruction on workplace related hazards and first aid		
13	Subjective assessment of specific protective measures (behaviour and regulations) related to infectious diseases	Protective measures and procedures are meant to reduce risks, e.g., infections. Do you believe this is provided by	Very vast extend–vast extent—party—small extend—very small extend	Self -constructed item(s)
		1. protective gloves, 2. protective clothing, 3. respiratory protective masks, 4. container for needle disposal (sharps container), 5. hygiene instruction, 6. maternity protection regulations, 7. hand and surface disinfection		
14	Frequency of contact to responsible specialist /offical after hazardous work situations	In the case of an occurrence of work related health hazards, how often did you contact	Always—often—sometimes—seldom–never	Self -constructed item(s)
		1. your supervisor, 2. a specialist for work safety, 3. the safety delegate of your unit, 4. a member of the staff council, 5. a hygiene specialist, 6. a company doctor, 7. an external institution /office		
		**“Twins”–Patient/Occupational Safety Culture**		
15	Direct supervisor’s expectations and actions promoting safety (PS/OS) *	Please indicate to what extent you agree with the following statements about your direct supervisor:	Strongly/partly agree–undecided–partly/strongly disagree	HSPSC (High 5s)
		1. My direct supervisor says a good word words when he sees that a task is done in accordance with established rules (standards and guidelines) 2. Whenever pressure builds up, my supervisor wants us to work faster 3. My direct supervisor seriously considers staff suggestions for improving PS/OS safety 4. My direct supervisor overlooks PS/OS safety problems that happen over and over		
16	Direct supervisors’ support for PS/OS *	Please indicate to what extend you agree with the following statements about your direct supervisor:	Strongly/partly agree–undecided–partly/strongly disagree	Adapted from HSPSC (High 5s)
		1. My direct supervisor provides a work climate that promotes PS/OS 2. The actions of my direct supervisor show that PS/OS is a top priority 3. My direct supervisor seems not interested in patient safety only after an adverse event happens		
17	Organisational learning–continuous improvement of PS/OS *	Please assess your unit:	Strongly/partly agree–undecided–partly/strongly disagree	HSPSC (High 5s)
		1. Mistakes have led to positive changes here 2. After we make changes to improve patient safety, we evaluate their effectiveness 3. We are actively doing things to improve patient safety PS/OS		
18	Hospital management’s support for PS/OS*	Please indicate to what extent you agree with the following statements about your hospital:	Strongly/partly agree–undecided–partly/strongly disagree	HSPSC (High 5s)
		1. The hospital management provides a work climate that promotes PS/OS 2. The actions of the hospital management show that of patient safety is top priority 3. The hospital seems interested in patient safety only after an adverse event happens		
19	Direct supervisor’s addressing of problems related to PS/OS- aspects *	My direct supervisor openly addresses problems concerning PS/OS in our hospital	Strongly/partly agree–undecided–partly/strongly disagree	Self-constructed item(s)
20	Direct supervisors’ increased focus on PS/OS *	My direct supervisor focuses more on PS/OS than a year ago	Strongly/partly agree–undecided–partly/strongly disagree	Adapted from *FTPS*
21	Direct supervisor’s attention to PS/OS *	It is important to my direct supervisor that our hospital pays great attention to PS/OS	Strongly/partly agree–undecided–partly/strongly disagree	Adapted from *FTPS*
22	Individual influence on PS/OS at the workplace *	Do you have an individual influence on how well PS/OS is implemented at the workplace?	Very vast extend–vast extent—party—small extend—very small extend	Self-constructed item(s)
23	General assessment of PS/OS at the workplace (global item) *	How would you evaluate the overall PS/OS in your unit?	Excellent–very good—acceptable—inadequate–insufficient	Self-constructed item(s)

Legend: * in all cases, PS and OS were assessed separately, i.e., by analogously verbalized items covering either PS(C) or OS(C). No 8, 9, 19–23: Single items. Abbreviations: ArbiK = Working Conditions in Hospitals Questionnaire, German [31]; HSPSC (High 5s project) [13]; FTPS = Safety Attitudes Questionnaire, German version [32]; OS = occupational safety; PS = patient safety.

**Table 2:** Individual knowledge and competencies related to patient and occupational safety aspects: Scores and items.

No. (Acronym)	Dimension	Items	Possible Range	Source
		**Patient Safety: Knowledge/Competencies**		
1 (PS-know) (score)	Knowledge about appropriate measures to avoid bladder catheter associated infections (multiple choice)	Which measures are appropriate to avoid catheter-acquired urinary tract infections in patients with indwelling catheters?	0–6 items appropriately answered	Self-constructed item(s)
		1. Indwelling urinary catheters should routinely be exchanged in strict intervals. (false) 2. Before an indwelling transurethral catheter is removed, it should intermittently be blocked by means of a clamp (so called “bladder-training”). (false) 3. The urine bag must hang freely without touching the floor and must be positioned lower than the patient’s bladder. (right) 4. Before working on the drainage system of the indwelling transurethral catheter, a hygienic and disinfection is necessary. (right) 5. Upon placing a long-term transurethral catheter, infection prophylaxis with an antibiotic is usually not necessary. (right) 6. During urine disposal, it is no problem if the drainage tap touches the receptacle. (false)		
2 (PS-comp) (score)	Competency to detect and treat infections of patients with indwelling bladder catheters (multiple choice)	In patients with indwelling transurethral catheters, it is particularly important to detect an infection and immediately start therapy. How is this accomplished?	0–3 items appropriately answered	Adapted from project *STOP Needlestick*
		1. Regular routine screening for asymptomatic bacteriuria. (right) 2. Bacteriuria screening indicated by clinical symptoms. (right) 3. Bladder wash-outs and insertion of fluids into the catheter as a means for infection prophylaxis. (false)		
		**Occupational safety: Knowledge/competencies**		
3 (OS-know) (scale)	Knowledge about post-exposure prophylaxis of Hepatitis-B- and C-virus and HIV (multiple choice)	After a needlestick injury, certain medications can prevent infection. For which infectious diseases is this an option?	0–3 items appropriately answered	Adapted from project *STOP Needlestick*
		1. hepatitis B (right) 2. hepatitis C (false) 3. HIV (right)		
4 (OS-comp) (score)	Competency for appropriate behaviour in the case of a needlestick injury (multiple choice)	You injured yourself with a used needle by sticking your fingertip. What should be done immediately?	0–5 items appropriately answered	“
		1. Put pressure on the affected area to stop the blood flow. (false) 2. Exercise pressure on hand and finger to increase the blood flow (“milk-out”) (right) 3. Rinse the affected area with hydrogen peroxide. (false) 4. Rinse the affected area with an antiseptic. (right) 5. Enlarge the injury with a scalpel. (false)		
5 OS-info (item)	Subjective information status to handle a needlestick injury appropriately	Do you feel sufficiently informed to deal with a “needlestick injury emergency?”	resp. cat. 0 = no 1 = yes	“

Legend: STOP Needlestick (Questionnaire used in the German research and evaluation project “STOP Nadelstich” [37].

**Table 3:** Sample characteristics (*n* = 547; original data; no imputation of missing values; valid cases of 407 nurses and 140 physicians).

Percent Values					Percent						Cases (Valid)					
					**Nurses**		**Physicians**		**Total**		**Nurses**	**Physicians**			**Total**	
Sex (female)					80.7		46.3		72.1		405	136			541	
Supervisor function (yes)					13.0		38.1		19.3		400	134			534	
Unlimited employment/permanent contract (yes)					94.3		29.3		78.1		402	133			535	
Employment contract > 75% (yes)					60.9		93.5		69.3		396	138			535	
**Mean values**	**Nurses**				**Physicians**						**Total**					
	**Mean**	**SD**	**Min-Max**	**N**	**Mean**	**SD**		**Min-Max**		**N**	**Mean**		**SD**	**Min-Max**		**N**
Age	39.9	11.2	20–63	384	37.6	8.7		24–65		129	39.3		10.6	20–65		513
Job tenure (years)	16.2	11.3	0–42	354	9.4	8.0		0–39		117	14.5		11.0	0–42		471
Employment in hospital (years)	14.3	9.7	0–41	343	7.5	6.8		0–38		112	12.6		9.5	0–41		455
Employment in current department (years)	8.9	8.1	0–37	336	3.6	4.4		0–21		112	7.6		7.7	0–37		448
Workload (hours/week)	33.9	8.5	8–41	244	41.0	6.3		19–70		107	36.0		7.8	8–70		351

Legend: Min-max = 0: indicating < 1 year. Abbreviations: SD = standard deviation.

**Table 4:** Individual knowledge and competencies related to patient and occupational safety aspects.

No.	Patient Safety–Knowledge and Competencies (Sum Score Means) (*n* = 410) *	Mean	SD	Min-Max	All Correct (%)
1 PS-know	Knowledge about appropriate measures to avoid bladder catheter associated infections (multiple choice; possible range 0–6)	4.14	1.00	1–6	13.9
2 PS-comp	Competency to detect and treat infections in patients with indwelling bladder catheters (multiple choice; possible range 0–3)	2.22	0.54	0–3	24.3
	**Occupational Safety–Knowledge and Competencies (Sum Score Means) (*n* = 547)**	**Mean**	**SD**	**Min-Max**	**All Correct (%)**
3 OS-know	Knowledge about post-exposure prophylaxis of hepatitis B- and C-virus and HIV infection (multiple choice; possible range 0–3)	1.97	0.62	0–3	13.7
4 OS-comp	Competency for appropriate behaviour in the case of a needlestick injury (multiple choice; possible range 0–5)	4.57	0.69	1–5	66.9
	**Occupational Safety–Subjective Information Status (Item Percent) (*n* = 547)**	**%**	**N**		
5 OS-info	Subjective information status to treat a needlestick injury appropriately (feeling well informed; possible answers: yes/no)	78.2	428		-

Legend: Abbreviations: SD = standard deviation. * excluded: Departments of Psychiatry/Psychotherapy and Dermatology, due to only little clinical relevance of the issue.
